# Genetic Causes of Rickets

**DOI:** 10.4274/jcrpe.2017.S008

**Published:** 2017-12-30

**Authors:** Sezer Acar, Korcan Demir, Yufei Shi

**Affiliations:** 1 Dokuz Eylül University Faculty of Medicine, Department of Pediatric Endocrinology, İzmir, Turkey; 2 King Faisal Specialist Hospital & Research Centre, Department of Genetics, Riyadh, Saudi Arabia

**Keywords:** Rickets, hereditary, genetic, vitamin D dependent, hypophosphatemic rickets

## Abstract

Rickets is a metabolic bone disease that develops as a result of inadequate mineralization of growing bone due to disruption of calcium, phosphorus and/or vitamin D metabolism. Nutritional rickets remains a significant child health problem in developing countries. In addition, several rare genetic causes of rickets have also been described, which can be divided into two groups. The first group consists of genetic disorders of vitamin D biosynthesis and action, such as vitamin D-dependent rickets type 1A (VDDR1A), vitamin D-dependent rickets type 1B (VDDR1B), vitamin D-dependent rickets type 2A (VDDR2A), and vitamin D-dependent rickets type 2B (VDDR2B). The second group involves genetic disorders of excessive renal phosphate loss (hereditary hypophosphatemic rickets) due to impairment in renal tubular phosphate reabsorption as a result of FGF23-related or FGF23-independent causes. In this review, we focus on clinical, laboratory and genetic characteristics of various types of hereditary rickets as well as differential diagnosis and treatment approaches.

## INTRODUCTION

Rickets is a disease of growing bone seen in children and adolescents due to deficiency in calcium, phosphate and/or vitamin D, leading to inadequate mineralization of osteoid tissue in the growth plate and bone matrix (1). The most frequent cause of rickets in Turkey, as well as in the rest of the world, continues to be nutritional vitamin D deficiency (1,2). Genetic causes of rickets (hereditary rickets) are rare: accounting for about 13% of total rickets (3).

They can be divided into two groups: vitamin D-dependent rickets which is caused by mutations either in enzymes involved in the vitamin D biosynthesis or vitamin D receptor (4), and hypophosphatemic rickets (HR) which is caused by impaired renal tubular phosphate reabsorption or transport due to genetic disorders associated with phosphatonins or phosphate co-transporters (5).

Calcium is one of the most common minerals in the body and it is mainly derived from dietary sources (6). It is essential for bone metabolism and various biological functions (6). While more than 99% of total calcium is stored in bone tissue as calcium-phosphate complex, less than <1% is distributed between intracellular and extracellular compartments (7). Of the <1% calcium outside bone tissue, 40% is bound to proteins, 9% is contained in ionic complexes and the remaining 51% is in the form of free Ca2+ ions that are the biologically active portion of body calcium (6,8). The ionized calcium balances the calcium pool in the intracellular-extracellular space and plays an important role in bone metabolism. This balance is achieved through the collective action of several hormones such as parathyroid hormone (PTH) and 1,25-dihydroxyvitamin D [1,25(OH)2D] and organs such as the kidney, bone and intestinal system (7,8). If serum calcium levels decrease, calcium-sensing receptors located on parathyroid cells mediate increased secretion of PTH, which binds to PTH 1 receptor (PTH1R, expressed in high levels in bone and kidney) to promote calcium resorption from bone and reabsorption from kidneys. PTH also activates 25-hydroxyvitamin D3-1α-hydroxylase, leading to increased 1,25(OH)2D synthesis, which promotes calcium absorption from intestines and reabsorption from proximal tubules of kidney (6,7,8).

Phosphorus is the most common anion in the human body. It is found in the form of inorganic phosphate and plays an important role in many biological processes such as bone mineralization, cell membrane integrity, nucleic acid and energy metabolism, signal transduction through phosphorylation of proteins and oxygen transport (9). In the adult male human, total body phosphorus is between 15 mol and 20 mol (12.0 g/kg), 80-90% of which is present in bone in the form of hydroxyapatite and the remaining 10-20% in soft tissue and extracellular spaces (9). Approximately two-thirds of dietary phosphate is absorbed via the sodium-dependent phosphate transporter 2B (NaPi-2b, encoded by the SLC34A2 gene), the major transporter that mediates phosphate reabsorption in the small intestine, predominantly in the jejunum. The expression of NaPi-2b is regulated by 1,25(OH)2D, which induces transcriptional up-regulation of NaPi-2b in the small intestine and low phosphate can activate 1α-hydroxylase in the kidney (10). Phosphate in the circulation can be taken up into cells for various biological activities or can be stored in the bone tissue. Approximately 85% of phosphate is reabsorbed by the sodium-dependent phosphate transporter 2A (NaPi-2a, encoded by the gene SLC34A1) and the sodium-dependent phosphate transporter 2C (NaPi-2c, encoded by the gene SLC34A3) both of which are expressed in the proximal tubules of the kidney (5,11). 1,25(OH)2D increases intestinal absorption of phosphate and tubular reabsorption, whereas PTH decreases tubular reabsorption of phosphate (TRP). In addition, other molecules that have phosphaturic effects, so-called phosphatonins, have significant impact on the balance of serum phosphate by reducing TRP (12,13).

Vitamin D is a group of biologically inactive, fat-soluble prohormones that exist in two major forms: ergocalciferol (vitamin D2) produced by plants in response to ultraviolet irradiation and cholecalciferol (vitamin D3) derived from animal tissues or 7-dehydrocholesterol in human skin by the action of ultraviolet rays present in sunlight with a wavelength of 270-290 nm (4). The main source of vitamin D is endogenous synthesis. Normally only 0.04% of 25-hydroxyvitamin D [25(OH)D] and 0.4% of 1,25(OH)2D are free in plasma, the remainder being tightly bound to either a vitamin D transporter protein (85-88%; high affinity) or albumin (12-15%; low affinity) (14). Both forms need two-step hydroxylation for activation. The first step occurs in the liver where vitamin D is hydroxylated to the minimally active 25(OH)D by hepatic 25-hydroxlase. The second step occurs mainly in the kidney where 25(OH)D is further hydroxylated by 1α-hydroxylase to become the biologically active hormone 1,25(OH)2D (calcitriol), which binds to its nuclear receptor vitamin D responsive (VDR) to regulate gene transcription through heterodimerization with one of three retinoid X receptor (RXR) isoforms (RXRα, RXRβ, RXRγ) and binds to cognate VDR elements (VDREs) in the promoter region of target genes (14,15). The renal synthesis of 1,25(OH)2D is stimulated by PTH and suppressed by calcium, phosphate and 1,25(OH)2D itself with renal 1α-hydroxylase being stimulated by PTH, hypophosphatemia or hypocalcaemia. Alternatively, 25(OH)D and 1,25(OH)2D may be catabolized to 24,25(OH)D and 1,24,25(OH)2D, respectively, through 24-hydroxylation by 25-hydroxyvitamin D 24-hydroxylase to maintain calcium homeostasis (4,14).

### 1. Vitamin D-Dependent Rickets

Disorders in the biosynthesis of vitamin D or its receptor activity result in vitamin D deficiency [vitamin D dependent rickets, type 1A (VDDR1A) and type 1B (VDDR1B)] or resistance [type 2A (VDDR2A) and type 2B (VDDR2B)]. All of them present similar clinical and biochemical manifestations of rickets such as findings related to hypocalcemia (irritability, fatigue, muscle cramps, seizures) and rickets (craniotabes, delayed closure of fontanelles, frontal bossing, enlarged wrists, bowed legs, short stature, and bone pain) (Table 1) (1,4).

### 1.1. Vitamin D-Dependent Rickets Type 1A

This disease, also called hereditary pseudo-vitamin D deficiency, was first described by Prader et al in 1961 as an autosomal recessive, persistent infantile rickets that responded to high dose vitamin D (16). Fraser et al (17) later reported that this condition was caused by lack of the 1-alpha hydroxylase enzyme. It is now defined as VDDR1A, (MIM 264700). VDDR1A occurs as a result of mutations in the CYP27B1 (cytochrome P450, family 27, subfamily B, polypeptide 1, MIM 609506) that encodes the 1-alpha hydroxylase enzyme (17,18). As a result, 25(OH)D cannot be converted to active 1,25(OH)2D, leading to clinical findings of rickets and vitamin D deficiency. To date, over 100 patients with 72 different mutations have been described in the Human Gene Mutation Database (HGMD, http://www.hgmd.cf.ac.uk/ac/index.php, accessed Nov 13, 2017) (4,14,19,20,21). Strikingly, in a genetically isolated population of French-Canadians in Quebec, the disease is found with the highest global incidence (1/2700) (4). The most commonly reported mutation in this region is 958delG, the “Charlevoix mutation”.

There is some genotype-phenotype correlation: milder phenotype is usually associated with mutations with residual enzyme activities (E189G, G102E and L343F) (22,23,24,25). Some milder cases may be missed and thus VDDR1A might be more common than is reported.

The disease is clinically similar to the phenotype of nutritional vitamin D-deficient rickets. The cases are usually normal at birth. However, growth retardation, skeletal deformities, muscle weakness, bone pain, muscle spasms and hypocalcemic convulsions may occur in the first year of life. The first observed findings in bone and joints include deformities such as craniotabes, metaphyseal enlargement, prominence of costochondral joints (rachitic rosary), delayed closure of the anterior fontanel, Harrison’s grooves and thoracic anomalies (1,26).

Similar to cases of nutritional rickets, typical cases with VDDR1A present with hypocalcemia, hypophosphatemia and increased serum levels of alkaline phosphatase (ALP) and PTH (Table 1). In contrast to nutritional rickets, levels of 25(OH)D are generally normal and 1,25(OH)2D are low (20). Some patients may be misdiagnosed as nutritional rickets and thus incorrectly treated with high dose vitamin D, leading to very high levels of 25(OH)D. Renal calcium excretion is low in these patients. In addition, hyperchloremic metabolic acidosis and hyperaminoaciduria secondary to PTH elevation can occur (4). Inappropriately normal 1,25(OH)2D levels in the presence of hypocalcemia can also be found in some patients with VDDR1A (20,27). Some cases might also be normocalcemic and a misdiagnosis of HR might be made before the detection of significantly elevated PTH levels (20).

Proper treatment of the disease includes administration of calcitriol, 1,25-dihydroxyvitamin D3 or alfacalcidol, 1 alpha-hydroxy-vitamin D3 in physiological doses (10-20 ng/kg/day, 2 doses), which will gradually improve clinical, biochemical and radiological findings (26). In addition, it is recommended to add 50-75 mg/kg/day of elemental calcium at the beginning of treatment. On follow-up, effective management should result in low-normal serum levels of calcium (8.5-9 mg/dL), normal phosphate levels and high-normal PTH values (4,26). High-normal levels of serum calcium might lead to hypercalciuria and subsequent development of nephrocalcinosis. Regular monitoring of 24-hour urinary calcium excretion and keeping the urine calcium excretion below 4 mg/kg/day is recommended (4,5,26). The degree of calciuria can also be assessed with spot urine calcium/creatinine ratios, for which varying normal ranges exist for different age groups: &0.8 mg/mg (≤6 months of age), &0.6 mg/mg (7-12 months), &0.53 mg/mg (1-3 years), &0.39 mg/mg (3-5 years), &0.28 mg/mg (5-7 years) and &0.21 mg/mg (>7 years ) (28).

### 1.2. Vitamin D Dependent Rickets Type 1B

VDDR1B (MIM 600081) is an extremely rare autosomal recessive disorder, due to 25-hydroxylase deficiency. This disease was first described in 1994 by Casella et al (29) in two Nigerian siblings of two and seven years old. Skeletal deformities compatible with rickets, hypocalcemia, hypophosphatemia, markedly elevated ALP and PTH, normal 1,25(OH)2D and low 25(OH)D levels were present. These siblings were diagnosed with 25-hydroxylase deficiency and showed clinical and laboratory improvement after high-dose vitamin D2 treatment. The gene encoding 25-hydroxylase (CYP2R1, MIM 608713) was described by Cheng et al (30) in 2003 and a homozygous CYP2R1 mutation (L99P) was identified in one of the first reported Nigerian siblings (31). Currently, only four CYP2R1 mutations are listed in the HGMD (accessed Nov 13, 2017). Apart from CYP2R1, there are five other cytochrome P450 enzymes (CYP27A1, CYP2J2/3, CYP3A4, CYP2D25 and CYP2C11) capable of catalyzing the initial 25-hydroxylation step (32). Indeed, a 20-month-old male patient has been described recently having hypocalcemic convulsions and rickets (33). His mother, maternal grandmother and aunt also have a history of hypercalcemic convulsion and skeletal deformities related with rickets in childhood. In all cases, hypocalcemia, hypophosphatemia, decreased 25(OH)D, markedly elevated ALP and PTH are present. Interestingly, a CYP2R1 mutation has not been found in this kin, suggesting that another gene may be involved in 25-hydroxylation. Calcitriol is the only choice of treatment for the disease (10-20 ng/kg/day, 2 doses).

### 1.3. Vitamin D Dependent Rickets Type 2A

**Figure 1:**
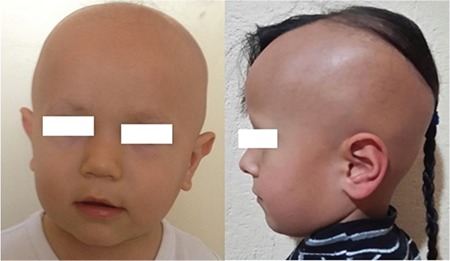
Near-total and partial alopecia in two children with VDDR2A (From the archives of Division of Pediatric Endocrinology, Dokuz Eylül University)

VDDR2A (MIM 277440), also known as hereditary vitamin D-resistant rickets, was first described by Brooks et al (34) in 1978 in a case who had skeletal findings suggesting rickets, short stature, hypocalcemia, elevated ALP, normal 25(OH)D, and very high 1,25(OH)2D. VDDR2A is an autosomal recessive disorder and is characterized by resistance to 1,25(OH)2D as a result of homozygous or compound heterozygous mutations in the vitamin D receptor gene (VDR, MIM 601769), which is located in 12q13.11 and consists of 11 exons. Patients with this disease usually present in infancy or early childhood, but patients with mild VDR defects may not be recognized until adolescence or adulthood (26). Clinical findings are similar to nutritional vitamin D deficiency or VDDR1A or VDDR1B except for high level of 1,25(OH)2D in VDDR2A (Table 1). Moreover, partial or total alopecia is present in many patients from birth or infancy (Figure 1) (35). The relationship between vitamin D and the hair follicle is not completely understood. However, VDR/RXRα heterodimer formation has been suggested to play an important role in the proliferation and differentiation of epidermal keratinocytes (36).

It is well known that active vitamin D mediates its biological functions by binding to its receptor VDR, which contains an N-terminal dual zinc finger DNA binding domain, a C-terminal ligand-binding domain and an extensive and unstructured region that links the two functional domains together (15). After binding of vitamin D, VDR forms a ternary structure with RXRα, which binds to a VDRE in the promoter region of vitamin D-regulated genes to initiate transcription (37,38). Currently, there are 65 different mutations listed in HGMD (accessed Nov 13, 2017). Inactivating mutations that affect any domain of VDR would lead to disease development. Mutations in the DNA binding domain that lead to complete loss of function result in severe clinical presentations accompanied by alopecia, whereas mutations in the ligand binding domain usually cause partial loss of VDR functions and a milder phenotype without alopecia (35,38). In addition to the genotype-phenotype relationship, the clinical presentation of the disease may improve with age. Serum levels of calcium, phosphate and ALP may gradually normalize in some pubertal cases and calcitriol/calcium treatment would be unnecessary (39,40,41). Intestinal calcium absorption has been shown to become less vitamin D-dependent after the end of puberty (40).

Hypocalcemia, hypophosphatemia, increased serum levels of ALP and PTH, and normal serum levels of 25(OH)D are usually found. Hypocalcemia, hypophosphatemia and increased PTH lead to activation of 1-alpha hydroxylase and inhibition of 24-hydroxylase. Therefore, low levels of 24,25(OH)2D and high levels of 1,25(OH)2D (300-1000 pg/mL, normal range: 15-90 pg/mL) are generally present (4,26).

High doses of oral calcitriol (1-6 μg/kg/day, 2 doses) and calcium (1-3 g/day elementary calcium) are the recommended treatment (26,39). Serum calcium, phosphate, ALP and PTH levels should be intermittently monitored and regular urine calcium excretion and renal ultrasonography are suggested because of the risk of nephrocalcinosis. Clinical presentation and response to treatment varies depending on the location of mutations in the VDR: patients with alopecia and nonsense mutations in the DNA-binding domain frequently exhibit a poor response to treatment (35,38). Treatment response may also be poor in patients without alopecia (42).

Long-term, high-dose intracaval/intravenous calcium (0.4-1.4 g/m2/day) treatment is also effective (38,43,44). After successful response to the treatment regimen, it is recommended to continue with high dose oral calcium (3.5-9.0 g/m2/day) (26,45). On the other hand, parenteral calcium therapy requires long-term hospitalization and may be associated with a number of complications such as cardiac arrhythmia, hypercalciuria, nephrocalcinosis, catheter related sepsis and extravasation of calcium (45,46). A case of VDDR2A without alopecia has been successfully treated with enteral administration of elemental calcium (calcium chloride) via gastric tube (47). Prolonged serum calcium deprivation might lead to secondary hyperparathyroidism and, if not managed properly, tertiary hyperthyroidism. Cinacalcet is reported to be effective in cases with VDDR2A and tertiary hyperparathyroidism (48,49).

### 1.4. Vitamin D Dependent Rickets Type 2B

VDDR2B (MIM 600785) is an unusual form of rickets due to abnormal expression of a hormone response element-binding protein that interferes with normal function of VDR. The disease was first described by Hewison et al (50) in 1993 in a patient with alopecia, skeletal abnormalities and biochemical features classically associated with VDRR2, but without VDR mutations (4). The similar clinical and genetic features were also found in more than 200 affected children from a rural area of southwest Colombia in 1995 (51). In contrast to VDDR2A, functions of VDR and VDR-RXR heterodimer formation are normal in VDDR2B (52). The main pathology is the overexpression of heterogeneous nuclear ribonucleoproteins (hnRNPs) C1 and C2 proteins, members of the hnRNP family, that prevent VDR-RXR heterodimer binding to VDRE (52,53). Without genetic testing, the differential diagnosis cannot be made between VDDR2A and VDDR2B (Table 1). The same treatment approaches for VDDR2A are used for patients with VDDR2B.

### 2. Hypophosphatemic Rickets

Hereditary HR is a group of rare, renal phosphate wasting disorders with a prevalence of 3.9 per 100,000 live births and differential diagnosis often requires genetic testing (54,55). It is characterized by renal phosphate wasting, leading to subsequent hypophosphatemia and bone mineralization defects such as rickets and osteomalacia. Hypophosphatemia and normal serum calcium are typical biochemical findings (55).

Serum levels of phosphate are maintained in the main by vitamin D and PTH. 1,25(OH)2D increases phosphate absorption from the intestine and suppresses the biosynthesis and secretion of PTH (5,56). PTH exhibits its phosphaturic effect by reducing the expression of NaPi-2a (SLC34A1) and NaPi-2c (SLC34A3) phosphate transporter in the renal tubules via PTH1R, a member of the G protein-coupled receptor family (5). In addition, several molecules [fibroblast growth factor 23 (FGF23), secreted frizzled related protein 4 (sFRP4), matrix extracellular phosphoglycoprotein, and FGF7], so-called phosphatonins, have been shown to reduce serum phosphate via direct inhibition of renal phosphate absorption in the proximal tubule (13). FGF23 and sFRP4 can also indirectly inhibit 25-OH vitamin D 1-α hydroxylase and thus intestinal phosphate absorption (57,58).

FGF23 is the most important phosphaturic agent and is produced from osteocytes and osteoblasts (57). There is a close relationship between serum phosphate and FGF23 levels. In response to elevated or decreased phosphate levels, serum FGF23 levels increase or decrease, respectively (5,58). FGF23 activates renal klotho/FGF receptor 1 (FGFR1) receptor heterodimers to inhibit renal phosphate reabsorption by down-regulation of NaPi-2a and NaPi-2c expression in the renal proximal tubules (58). FGFR3 and FGFR4 are also involved in mediating FGF23 activities (59). Klotho, a transmembrane protein, is required for FGF23 function and klotho knockout mice exhibit extremely high levels of serum FGF23, most likely due to end-organ resistance to FGF23 (60,61). In addition, FGF23 inhibits 25-OH vitamin D 1-α hydroxylase and activates 25-OH vitamin D 24-hydroxylase, resulting in decreased 1,25(OH)2D and increased 24,25(OH)2D levels (62).

Another molecule that plays a role in phosphate regulation is sodium-hydrogen exchanger regulatory factor 1 (NHERF1) (58). NHERF1 has been shown to have two different effects on phosphate reabsorption in the proximal tubules. The first is to bind to PTH1R to reduce the effect of PTH-induced cAMP synthesis and the second is to increase the activation of NaPi-2a by interacting with C-terminal region of the protein (58,62).

Serum phosphate levels normally vary according to age, which needs to be carefully considered when assessing whether hypophosphatemia is present or not. Normal ranges of serum phosphate are 4.8-8.2 mg/dL for 0-5 days of age, 3.8-6.5 mg/dL for 1-3 years of age, 3.7-5.6 mg/dL for 4-11 years of age, 2.9-5.4 mg/dL for 12-15 years of age and 2.7-4.7 mg/dL for 16-19 years of age (27). In addition to hypophosphatemia, decreased TRP, normal or mildly elevated serum levels of PTH and markedly elevated serum levels of ALP are typically detected. In a study comparing serum levels of ALP and PTH in HR, VDDR and nutritional rickets, the highest serum levels of PTH and ALP have been found in patients with VDDR and the lowest levels in patients with HR (63).

Renal phosphate excretion can be evaluated using various parameters. The most widely used is the TRP defined by the formula: 1-(urine phosphate x serum creatinine) / (serum phosphate x urine creatinine). Various lower limits for TRP are generally used in daily practice ranging from 75-85%. However, in the presence of hypophosphatemia, fractional excretion of filtered phosphate should be less than 5% (TRP >95%) (64). The ratio of tubular maximum reabsorption rate of phosphate per glomerular filtration rate (TmP/GFR) is a superior method for assessing phosphaturia, which can be assessed via the nomogram of Walton and Bijvoet or can be calculated as shown below:

For TRP ≤86%: TmP/GFR= TRP x serum phosphate

For TRP >86%: TmP/GFR= (0.3 x TRP) / [1-(0.8 x TRP)] x serum phosphate

Low TmP/GFR values in the setting of hypophosphatemia points to renal phosphate wasting (65). The normal ranges of TmP/GFR (mg/dL) vary with age: Birth, 3.6-8.6; 3 months of age, 3.7-8.25; 6 months of age, 2.9-6.5; 2-15 years of age, 2.9-6.1, and the normal adult range for TmP/GFR is 2.2 to 3.6 mg/dL (66).

Laboratory findings such as normal serum calcium, low serum phosphate and elevated serum ALP and PTH may not always be diagnostic of HR. These can also be seen in rickets (especially in stage 2) associated with vitamin D deficiency or disorders of vitamin D biosynthesis (20). The distinctive finding is that PTH is significantly higher in vitamin D-related rickets, whereas normal/mildly elevated PTH is expected in HR (26). To date, a variety of genetic causes leading to HR have been identified (Table 2) (5,58,62). Some of these genetic defects lead to an increase in serum FGF23 levels (FGF23-related or -dependent HR), while others affect phosphate transporters which does not affect serum FGF23 levels (FGF23-independant HR). Laboratory characteristics of several types of HR are summarized in Table 3.

### 2.1. FGF23-Related Hypophosphatemic Rickets

**2.1.1. X-linked Dominant Hypophosphatemic Rickets**

X-linked dominant HR (XLDHR, MIM 307800) is the most common type of HR with an incidence of approximately 1 in 20000 live births and is caused by inactivating mutations of *PHEX* (phosphate regulating gene with homologies to endopeptidases on the X chromosome, MIM 307800) (55,67). XLDHR affects both genders equally in terms of disease severity as a result of random X-inactivation in girls (62). Skeletal findings of the disease frequently appear in the late infantile period and are especially evident by the effect on body weight in the period after starting to walk (5). *PHEX* encodes a membrane endopeptidase, which is expressed in mature osteoblasts and odontoblasts, and plays a role in down-regulation of FGF23 expression (68). Therefore *PHEX* mutations would lead to increased serum levels of FGF23 (69). Currently, there are 423 *PHEX* mutations listed in HGMD (accessed Nov 13, 2017).

In the Turkish population, *PHEX* mutation is also the most common cause of HR, accounting for 87% cases (55,70,71). *De novo* mutations are frequent and more often occur in female patients, likely resulting from mutagenesis of the X chromosome in paternal germ cells (70).

Typical clinical findings include short stature, wrist enlargement, rachitic rosary, bowed legs, frontal bossing, dental abscess and bone pain in children. Osteomalacia, bone pain, dental abscess and spinal canal stenosis are typical presentation in adult patients. Laboratory findings include low serum levels of phosphate, decreased TRP, normal/mildly elevated PTH and high levels of ALP with normal calcium and 25(OH)D, and inappropriately normal or low serum 1,25(OH)2D levels (Table 3). These clinical and laboratory findings suggest HR but confirmation of diagnosis requires genetic confirmation of *PHEX* mutations.

**2.1.2. Autosomal Dominant Hypophosphatemic Rickets**

Autosomal dominant HR (ADHR, MIM 193100) is caused by gain-of-function mutations in the proteolytic cleavage domain of FGF23 (R176XXR179, MIM 605380). Mutations that alter the arginine (R) residue at the position 176 or 179 would render the protein resistant to proteolytic cleavage and lead to increased serum levels of FGF23 and its activity, resulting in hypophosphatemia (61,71,72). It is less common than XLHR and 16 different mutations are reported in HGMD (accessed Nov 13, 2017).

ADHR exhibits similar clinical and laboratory findings as XLHR and also needs genetic testing for diagnosis. Differences in the age of onset, severity and a waxing and waning course of phosphate wasting (renal phosphate wasting can be spontaneously normalized) is related to serum FGF23 levels (73,74). This led to the discovery that iron deficiency is an environmental trigger, which stimulates FGF23 expression and thus hypophosphatemia in ADHR (75,76,77).

**2.1.3. Autosomal Recessive Hypophosphatemic Rickets**

**2.1.3.1. Autosomal Recessive Hypophosphatemic Rickets Type 1**

ARHR type 1 (ARHR1, MIM 241520) is due to inactivating homozygous mutations in the DMP1 gene (dentin matrix acidic phosphoprotein 1, MIM 600980) (78). DMP1 is an extracellular matrix protein expressed in osteoblasts and osteocytes and acts in the inhibition of FGF23 expression (62,68). Inactivating mutations of DMP1 result in an increase in serum FGF23 levels and thus leads to HR. Clinical, laboratory and radiological findings are similar to those of XLHR and ADHR. There are 9 different mutations listed in the HGMD (accessed Nov 13, 2017). DMP1 knockout mice have displayed increased serum levels of FGF23, hypophosphatemia, skeletal and dental anomalies and osteomalacia (79). Unlike other HR types, osteosclerosis in the base of skull and calvarial bones may occur (62). Haploinsufficiency has been reported in heterozygous carriers: mild hypophosphatemia, low TRP and focal osteomalacia, without typical skeletal deformities of rickets (80).

**2.1.3.2. Autosomal Recessive Hypophosphatemic Rickets Type 2**

ARHR type 2 (ARHR2, MIM 613312) is caused by inactivating homozygous mutations in ENPP1 (ectonucleotide pyrophosphatase/phosphodiesterase 1, MIM 173335) (81). Interestingly, the majority of ENPP1 mutations (49 mutations) have been reported in patients with idiopathic infantile arterial calcification or generalized arterial calcification of infancy, which is an autosomal recessive disorder and characterized by calcification of the internal elastic lamina of muscular arteries and stenosis due to myointimal proliferation (82). There are only eight mutations reported in patients with HR (HGMD, accessed Nov 13, 2017), suggesting a different pathway is involved in the generation of ARHR2 (83).

By generating inorganic pyrophosphate (PPi), ENPP1 plays an important role in the regulation of pyrophosphate levels, bone mineralization and soft tissue calcification. The mineral accumulation in the bones is determined by the ratio of phosphate and PPi that is balanced by ENPP1 (84). Enpp1 knockout mice show altered bone development and an increase in FGF23 expression (84). ENPP1 mutations increase serum levels of FGF23. However, the mechanism of FGF23 elevation caused by ENPP1 mutation is not completely understood (82,83,84).

**2.1.4. Hypophosphatemic Rickets with Hyperparathyroidism**

HR with hyperparathyroidism (MIM 612089) is a very rare disease caused by a balanced translocation with breakpoints at 9q21.13 and 13q13.1, which is adjacent to the KL gene (85). Its product, alpha-Klotho, is implicated in aging and regulation of FGF signaling and calcium homeostasis (86). The translocation result in increased serum α-klotho, FGF23 levels and β-glucuronidase activity (85). The disease is characterized by hypophosphatemia and elevated serum PTH levels, with inappropriate renal phosphate wasting (85). Increased levels of FGF23 lead to decreased TRP, hypophosphatemia and rickets. Hyperparathyroidism due to diffuse parathyroid hyperplasia results in increased levels of PTH. It is not clear whether increased levels of α-klotho cause parathyroid hyperplasia. PTH levels in this disease are much higher compared to other causes of HR and are comparable with those in VDDR. Klotho knockout mice, deficient for α-klotho, display a phenotype comparable with human ageing and are characterized by a mild hypercalcemia, hyperphosphatemia, increased levels of serum 1,25(OH)2D, decreased PTH and bone abnormalities such as increased metaphyseal trabecular bone mass and soft tissue calcifications, which are different from the phenotype caused by the translocation [hypophosphatemia, high PTH, and normal 1,25(OH)2D7] (87,88). Treatment includes calcitriol with oral phosphate supplementation.

**2.1.5. Other Genetic Causes**

**2.1.5.1. Osteoglophonic Dysplasia**

Osteoglophonic dysplasia (MIM 166250) is caused by heterozygous gain-of-function mutations in FGFR1 (MIM 136350), a rare autosomal dominant disorder characterized by craniosynostosis, rhizomelic short stature, maxillary hypoplasia, depressed nasal bridge, mandibular pragmatism, dental anomalies, tower-shaped skull, vertebral anomalies and bone mineralization defects (metaphyseal radiolucent changes) (89). High levels of serum FGF23, low levels of serum phosphate and 1, 25(OH)2D, and low TRP are present in some patients (89). Increased FGF23 leads to renal phosphate wasting, hypophosphatemia and deterioration of bone mineralization. It has been suggested that FGF23 production is stimulated from bone tissue due to the effect of activating mutations in FGFR1 (5). Among 197 mutations in FGFR1, only three are reported in patients with osteoglophonic dysplasia (HGMD, accessed Nov 13, 2017).

**2.1.5.2. McCune-Albright Syndrome**

McCune-Albright Syndrome (MAS, MIM 174800) is caused by post-zygotic activating mutations in the Gsα subunit of G proteins (encoded by GNAS, MIM 139320), leading to a mosaic distribution of cells bearing constitutively active adenyl cyclase activity. The disease is characterized by the classic triad of polyostotic fibrous dysplasia, cafe-au-lait skin pigmentation and peripheral precocious puberty, but is clinically heterogeneous and usually include hyperfunctional endocrinopathies such as thyrotoxicosis, pituitary gigantism and Cushing syndrome due to autonomous hormonal hyper-production (90). There is an association between fibrous dysplasia of bone tissue and increase in serum FGF23 level. TRP is decreased in 50% of cases (91). Therefore, hypophosphatemic rickets/osteomalacia can be seen in these patients. More than 250 mutations are listed in the HGMD (accessed Nov 13, 2017) and most of them (221 inactivating mutations) are found in patients with resistance to PTH (pseudohypoparathyroidism or Albright hereditary osteodystrophy, which is different from the disease). In all patients reported to date, there are only two activating mutations (p.R201H or p.R201C and p.T55A) listed in the HGMD (accessed Nov 13, 2017) that is associated with McCune-Albright Syndrome.

**2.1.5.3. Raine Syndrome**

Raine syndrome (MIM 259775) is an autosomal recessive disorder first described in 1989 by Raine et al (92) in a case with generalized osteosclerosis of the periosteal bone formation and severe craniofacial dysmorphology. The disease is caused by mutations in the FAM20C (family with sequence similarity 20, member c, also called dentin matrix protein 4 DMP4; MIM 611061) and was initially reported to be lethal (93). Non-lethal cases have since been found (94). FAM20C is mainly expressed in osteoblasts, odontoblasts and ameloblasts in skeletal and dental tissues and is a novel FGF23 regulator (95,96). Increased renal phosphate loss and hypophosphatemia due to increased serum FGF23 levels have been reported in Raine’s syndrome (97,98,99). HR has been observed in FAM20C knockout mice (96). FAM20C can suppress FGF23 production by enhancing DMP1 expression and its inactivation causes FGF23-related hypophosphatemia by decreasing transcription of DMP1, resulting in increased FGF23 levels in patients with Raine’s syndrome (98). There are 22 mutations listed in the HGMD (accessed Nov 13, 2017).

**2.1.5.4. Opsismodysplasia**

Opsismodysplasia (OPSMD, MIM 258480) is a rare skeletal dysplasia involving delayed bone maturation first described by Zonana et al (100) in 1977 and later defined by Maroteaux et al (101) in 1982. It is an autosomal recessive disease and caused by mutations in the INPPL1 gene (inositol polyphosphate phosphatase-like 1, MIM 600829) (102). Clinical signs observed at birth include short limbs, small hands and feet, relative macrocephaly with a large anterior fontanelle and characteristic craniofacial abnormalities such as a prominent brow, depressed nasal bridge, a small anteverted nose and relatively long philtrum. Abdominal protrusion, abnormalities of the extremities, progressive bone demineralization, delayed bone maturation and hypotonia are commonly reported (103). The main radiological features are severe platyspondyly, short long bones including squared metacarpals, delayed epiphyseal ossification, and metaphyseal flaring and cupping (103). In addition to these clinical and radiological findings, increased renal phosphate excretion and HR have been reported by Zeger et al (104). The serum level of FGF23 was high in one of the two patients at three years of age. Currently, there are 26 mutations listed in the HGMD (accessed Nov 13, 2017).

**2.1.6. Treatment of FGF23-related Hypophosphatemic Rickets**

There is no difference in the management of XLHR, ADHR, ARHR and other rare genetic causes of HR. It is a lifelong treatment of phosphate and calcitriol replacement to restore bone mineralization and improve skeletal deformities. Calcitriol is recommended at doses ranging from 25 to 70 ng/kg/day (2 doses) and elemental phosphate at 30 to 70 mg/kg/day (4-6 doses) (26). The main goal of treatment is to achieve low-normal serum phosphate and high-normal serum ALP levels (105). Treatment should not attempt to normalize serum phosphate levels by giving aggressive phosphate therapy as this might lead to side effects such as diarrhea, secondary hyperparathyroidism, increased FGF23 synthesis, nephrocalcinosis and renal insufficiency (105). In addition, serum phosphate levels should not be used alone in evaluating response to treatment, due to rapid fluctuations in serum levels. Therefore, reduction in ALP levels, improvement in clinical findings and growth velocity after treatment are more useful indicators in assessing treatment response. Traditional calcitriol and phosphate therapy improves bone mineralization, skeletal findings of rickets and growth rate. However, despite these treatments, skeletal deformities may persist to varying degrees in some patients (105).

Phosphate salts (sodium phosphate, potassium phosphate) are generally used for phosphate replacement. It can be given in tablet or solution form both of which are equally effective. Tablet form (Phosphate-Sandoz®) contains a high dose of phosphate supplement, consisting of sodium phosphate monobasic. Each tablet provides elemental phosphate 500 mg (16.1 mmol phosphate), sodium 469 mg (20.4 mmol Na+), potassium 123 mg (3.1 mmol K+) and citric acid-anhydrous 800 mg. “Joulie’s solution” can be used for children if the tablet form is not available. Prepared with 136 g of dibasic sodium phosphate, 58.8 g phosphoric acid and 1000 mL of distilled water, 1 mL of this solution contains 30.4 mg of elemental phosphate (106). More frequent dividing of phosphate dose avoids a profound drop in post-dose serum phosphate levels and reduces the frequency of diarrhea, the most common side effect of this treatment.

Patients should be monitored for clinical, anthropometric and laboratory characteristics at three month intervals. Laboratory assessments include serum calcium, phosphate, ALP and PTH levels, as well as urinary calcium and creatinine for hypercalciuria. In addition, renal ultrasonography should be performed annually, before and after treatment, to monitor the development of nephrocalcinosis (105). Skeletal X-ray is recommended to be performed annually before treatment and during treatment for monitoring of skeletal findings (5).

The dosage of calcitriol should be adjusted according to serum levels of PTH and the urine calcium/creatinine ratio. The main goal is to suppress PTH, maintain serum calcium in the normal range and prevent hypercalciuria. Twenty-four hours of urinary calcium excretion above 4 mg/kg/day indicates increased calcium excretion (hypercalciuria) (26). In addition, the ratio of calcium to creatinine in the spot urine can be used. The normal range varies with age: ≤6 months of age, <0.8; 7-12 months of age, <0.6; 1-3 years of age, <0.53; 3-5 years of age, <0.39; 5-7 years of age, <0.28; >7 years of age, <0.21 (28). In the presence of hypercalciuria, it is necessary to reduce calcitriol dosage. The evening dosage of calcitriol should be higher in order to suppress increased secretion of PTH at night (26).

There is a close relationship between high dose phosphate therapy and the development of nephrocalcinosis (107,108). The frequency of nephrocalcinosis in HR patients after calcitriol and phosphate combined therapy is between 33% and 80%, and usually occurs within the first 3-4 years of treatment (105,107,108,109). However, long-term follow-up of cases with nephrocalcinosis has been reported to have no significant impairment on renal function (110). On the other hand, long-term, high-dose phosphate therapy may result in secondary and tertiary hyperparathyroidism (105,111,112,113). Cinacalcet can be used in the treatment of tertiary hyperparathyroidism in children with HR (111). In brief, oral phosphate should be given at the lowest dose that is sufficient to improve rickets and patients should be monitored for the development of hyperparathyroidism and nephrocalcinosis.

Conventional treatment should gradually improve biochemical and skeletal abnormalities, however mild or moderate skeletal deformities may persist in some patients. For these patients, some devices, such as braces, are suggested to correct leg bowing. If such devices are not tolerated, surgical correction can be considered. In children younger than 10 years with XLHR, femoral and tibial hemiepiphysiodesis are recommended to correct lower extremity deformities, which is a relatively minor surgical procedure to allow appropriate growth (114). For children older than 10 years of age, osteotomy is suggested, a surgical procedure in which a surgeon removes a wedge of bone near a damaged joint (26).

Short stature is one of the major findings in the diagnosis of HR patients. With appropriate calcitriol and phosphate treatment, the skeletal and biochemical findings should improve and an increase in height velocity should be achieved. However, some patients with XLHR do not achieve the desired height velocity despite appropriate treatment (115,108). It is suggested that this may be related to delayed treatment or deficit in GH secretion (115,116). Recombinant human growth hormone (rhGH) treatment, especially in the pre-pubertal period, has been demonstrated to significantly increase height velocity and positively contributes to final height in these patients (117,118,119).

Recent progress in treatment has focused on the pathogenesis of HR. It has been shown that pharmacological inhibition of FGF receptor signaling ameliorates FGF23-mediated HR using NVP-BGJ398, a novel, selective, FGFR inhibitor that inhibits FGFR1, FGFR2, and FGFR3 with IC50 of 0.9 nM, 1.4 nM, and 1 nM, respectively (120). Similar results have been achieved using anti-FGF23 antibody (KRN23), a human monoclonal KRN23 (121). In a study of 28 adults with XLHR who received monthly KRN23, a significant increase in serum phosphate, 1,25(OH)2D and maximum renal tubular threshold for phosphate reabsorption (TmP/GFR) has been observed after four or twelve months of treatment (121). The half-life is 8-12 days after intravenous administration and longer (13-19 days) after subcutaneous administration. The serum levels of phosphate remained higher than baseline level for four weeks (122,123). Therefore, it is recommended that KRN23 should be given at four weekly intervals. Finally, phase III studies of KRN23 in adults and children are still ongoing.

### 2.2. Hypophosphatemic Rickets Accompanied by Hypercalciuria (FGF23-independent Rickets)

**2.2.1. Hereditary Hypophosphatemic Rickets with Hypercalciuria**

Hereditary HR with hypercalciuria (HHRH, MIM 241530) is an autosomal recessive disease caused by inactivating mutations in the SLC34A3 (solute carrier family 34, member 3, also known as NaPi-2c, MIM 609826) (124). SLC34A3 plays a role in phosphate reabsorption in the kidney and its mutation results in increased renal phosphate loss and subsequent hypophosphatemia (5). FGF23 is not involved in the disease. The decrease in serum phosphate promotes biosynthesis of 1,25(OH)2D, which leads to increase in the absorption of intestinal calcium, suppressed PTH and development of hypercalciuria and nephrocalcinosis. Diagnosis can be made based on skeletal findings of rickets, hypophosphatemia, hypercalciuria and nephrolithiasis (124,125). There are 33 mutations listed in HGMD (accessed Nov 13, 2017) and genotype-phenotype correlation has not yet been established (125,126,127). Increased renal phosphate wasting, mild hypophosphatemia, increased 1,25(OH)2D and hypercalciuria without metabolic bone disease, can be present in patients with heterozygous SLC34A3 mutations, indicating haploinsufficiency (124).

Oral phosphate alone is sufficient for patients with HHRH in contrast to patients with XLHR, ADHR or ARHP, who are usually treated with high doses of alphacalcidol or calcitriol and multiple daily doses of oral phosphate, low-sodium diet and hydration are recommended for the disease (5,26). The response to treatment is excellent. Phosphate treatment results in a decrease in serum levels of calcitriol and, consequently, urinary calcium excretion gradually returns to normal. The use of calcitriol is contradictory and harmful because it can increase hypercalciuria.

**2.2.2. Hypophosphatemic Rickets with Nephrolithiasis and Osteoporosis Type 1**

SLC34A1 (solute carrier family 34, member 1, MIM 182309) encodes NaPi-2a, which plays an important role in phosphate reabsorption from proximal tubules and is down-regulated by PTH and FGF23 (128). Inactivating mutations in SLC34A1 can cause three different diseases: HRs with Nephrolithiasis and Osteoporosis type 1 (NPHLOP1, MIM 612286) (129,130), Fanconi Renotubular Syndrome type 2 (FRTS2, MIM 613388) (131) and Infantile Hypercalcemia type 2 (HCINF2; MIM 616963) (132). NPHLOP1 was originally reported as an autosomal-dominant disease. However, multiple groups later questioned a single heterozygous mutation in the pathogenesis of the disease (131,133,134). The initial cases caused by heterozygous SLC34A1 mutations are probably represent a milder phenotype characterized by increased renal phosphate wasting, hypercalciuria, osteoporosis and nephrolithiasis in adults. Currently, there are 25 different mutations listed in the HGMD (accessed Nov 13, 2017).

Similar to HHRH, NPHLOP1 is characterized by hypophosphatemia and decreased renal phosphate absorption with an appropriate elevation in serum 1,25(OH)2D. Laboratory findings include decreased TRP, hypophosphatemia, hypercalcemia, elevated serum 1,25(OH)2D, decreased serum PTH, hypercalciuria and nephrocalcinosis.

The original patients with FRTS2 were adults with clinical features of increased renal phosphate and other substance wasting (without loss of bicarbonate) and significantly increased 1,25(OH)2D leading to severe skeletal deformities (HR in children and osteomalacia in adults), bone pain, marked hypercalciuria, glycosuria, generalized aminoaciduria and tubular proteinuria without renal tubular acidosis (135).

HCINF2 is characterized by severe hypercalcemia with failure to thrive, vomiting, dehydration and medullary nephrocalcinosis. Laboratory findings include decreased TRP, hypophosphatemia, hypercalcemia, elevated 1,25(OH)2D, suppressed PTH, hypercalciuria, nephrocalcinosis, hyperuricosuria and low-molecular-weight proteinuria (136).

The main pathogenesis of all three diseases is increased phosphate wasting due to inactivated phosphate cotransporter NaPi-2a in the proximal tubules. They should be considered as one disease with different clinical presentations, probably caused by differences in severity of mutations. The mechanism for renal tubulopathy is unclear at present.

Treatment is the same as in HHRH. Oral phosphate replacement will result in improvement in bone pain, muscle strength and radiologic signs of rickets, with normalization of urinary calcium excretion and significant decrease in 1,25(OH)2D. However, the glomerular filtration rate, serum uric acid levels and rate of urinary excretion of glucose, protein and amino acids will remain unchanged.

**2.2.3. Hypophosphatemic Rickets with Nephrolithiasis and Osteoporosis Type 2**

HRs with Nephrolithiasis and Osteoporosis type 2 (Nephrolithiasis/osteoporosis, hypophosphatemic, 2, NPHLOP2, MIM 612287) is an autosomal dominant disease caused by mutations in the SLC9A3R1 (MIM 604990). It encodes NHERF1, an adaptor protein that regulates several G protein-coupled receptors, including the PTH1R (58,137). It regulates phosphate reabsorption in the renal proximal tubules by binding to renal phosphate transporter NaPi-2a to maintain correct expression at the apical domain of proximal tubular cells and PTH1R leading to a decrease in PTH-induced cAMP synthesis and phosphate transport (128,138). Mutations in the NHERF1 result in reduced NaPi-2a expression and hypophosphatemia due to increased renal phosphate loss. Characteristic clinical features include hypophosphatemia, hypercalcemia, elevated serum levels of 1,25(OH)2D, hypercalciuria, decreased TRP or low TmP/GFR value and nephrolithiasis, which cannot be distinguished from HHRH or NPHLOP1 without molecular testing. Serum levels of PTH and FGF23 are normal. Osteopenia has been demonstrated in patients with NHERF1 mutations, although rickets has not yet been reported, probably reflecting late-onset and milder phenotype caused by the gene mutation. There are only four different mutations listed in the HGMD (accessed Nov 13, 2017).

**2.2.4. Dent Disease**

Dent disease can be divided into type 1 and type 2. Dent disease 1 (MIM 300009, also known as X-linked nephrolithiasis, X-linked nephrolithiasis type 2 (NPHL2), X-linked recessive nephrolithiasis with renal failure, or X-linked recessive nephrolithiasis type 1 (NPHL1), MIM 310468) is an X-linked recessive disease caused by mutations in the CLCN5 gene which encodes chloride voltage-gated channel 5 (MIM300008) (139). It is characterized by proximal tubular dysfunction and 30-80% of patients can progress to chronic kidney disease or renal failure: low molecular weight proteinuria, hypercalciuria, glycosuria, phosphaturia, aminoaciduria, uricosuria, hematuria and nephrocalcinosis (140,141,142). More than 259 different CLCN5 mutations are listed in the HGMD (accessed Nov 13, 2017). The presence of hypophosphataemic rickets in Dent disease is variable from 30-50% in patients from US and UK, to rare in Japanese patients (142,143,144). Clinical presentations and CLCN5 mutations are heterogeneous and there is no genotype-phenotype correlation.

Dent disease 2 (MIM 300555, or Lowe syndrome or oculocerebrorenal syndrome, MIM 309000) is also an X-linked recessive disease caused by mutations in the OCRL gene (MIM 300535) which encodes inositol polyphosphate-5-phosphatase (145). Clinical features are similar to Dent disease 1 and genetic testing is required to distinguish between them. There is a broad phenotypic spectrum of OCRL mutations and Dent disease 2 may be a mild variant of Lowe syndrome characterized by hydrophthalmia, cataract, mental retardation, HR, amino aciduria, proteinuria and phosphaturia (146).

There are 245 different OCRL mutations listed in the HGMD (accessed Nov 13, 2017). Approximately 50-60% of cases with Dent disease have CLCN5 mutations, 15-20% have OCRL mutations and the remaining cases have no detectable mutation (140,146). Patients usually respond well to oral phosphate for the treatment of hypophosphatemia. In addition, some patients may need calcitriol, but it should be carefully used as it may increase urinary calcium excretion. A sodium-restricted diet to reduce urinary calcium excretion may be useful.

## Conclusion

Calcium and phosphate, which play important roles in bone mineralization, are regulated by various molecules such as PTH, 1,25(OH)2D and FGF23. Nutritional vitamin D deficiency is the most common cause of rickets due to low vitamin D in breast milk, social and economic conditions that prevent access to vitamin D from other sources, or climatic conditions preventing adequate ultraviolet light exposure. Various genetic causes of rickets should be considered to avoid delay in diagnosis and treatment. Rickets caused by calcium deficiency should also be considered, which usually occurs among older toddlers and children due to low dietary calcium intake. Although clinical presentations are usually similar, differential diagnosis of different types of rickets such as nutritional and VDDR (VDDR1A, VDDR1B, VDDR2A and VDDR2B) can be made by examining serum levels of 25(OH)2D and 1,25(OH)2D, and their responses to treatment (calcium, vitamin D or calcitriol) (Table 1).

The genetic causes of HR can be divided into two groups: FGF23-dependent and FGF23-independent groups (Table 2). The most common genetic cause of HR is XLDHR resulting from *PHEX* mutations. Although clinical presentations are similar, differential diagnosis between these two groups can be made by serum FGF23 levels. However, diagnosis of individual diseases within each group often require molecular testing to confirm diagnosis. The current treatment for FGF23-dependant HR is oral phosphate replacement and calcitriol which have potential treatment complications such as calciuria and nephrocalcinosis. Recent progress of targeted therapy against FGF23-mediated HR (NVP-BGJ398 and KRN23) has produced promising results and may offer better therapeutic outcome in the future. In the FGF23-independent HR group, hypercalciuria and nephrolithiasis are major clinical findings and oral phosphate replacement alone is sufficient in the treatment. Furthermore, there are some HR patients whose genetic defects remain to be identified.

## Figures and Tables

**Table 1:**
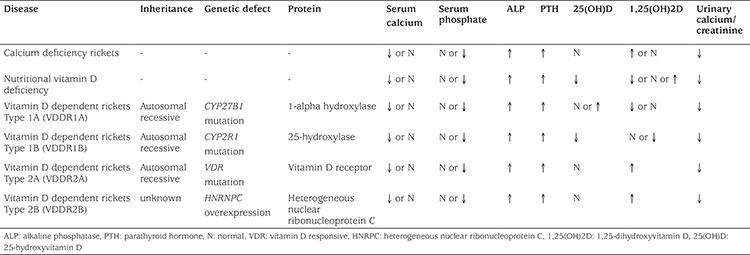
Laboratory characteristics of rickets associated with vitamin D metabolism

**Table 2:**
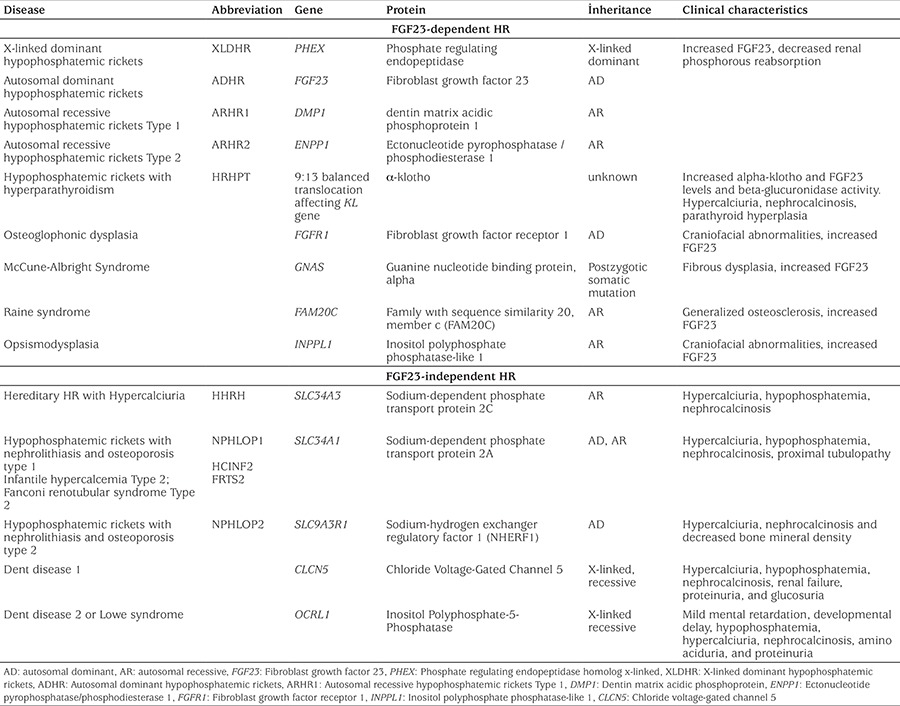
Genetic causes of hypophosphatemic rickets

**Table 3:**
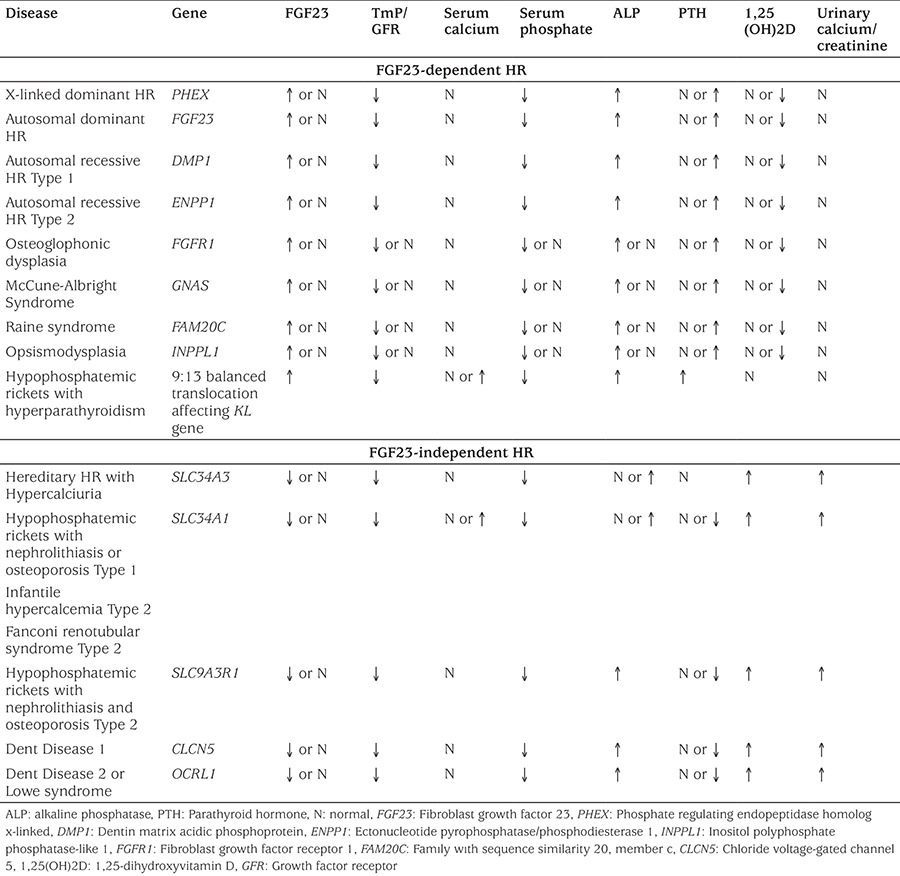
Laboratory characteristics of genetic causes of hypophosphatemic rickets

## References

[1] Misra M, Pacaud D, Petryk A, Collett-Solberg PF, Kappy M, Drug and Therapeutics Committee of the Lawson Wilkins Pediatric Endocrine Society (2008). Vitamin D deficiency in children and its management: review of current knowledge and recommendations. Pediatrics.

[2] Hatun Ş, Ozkan B, Bereket A (2011). Vitamin D deficiency and prevention: Turkish experience. Acta Paediatr.

[3] Beck-Nielsen SS, Brock-Jacobsen B, Gram J, Brixen K, Jensen TK (2009). Incidence and prevalence of nutritional and hereditary rickets in southern Denmark. Eur J Endocrinol.

[4] Miller WL (2017). Genetic disorders of Vitamin D biosynthesis and degradation. J Steroid Biochem Mol Biol.

[5] Bastepe M, Jüppner H (2008). Inherited hypophosphatemic disorders in children and the evolving mechanisms of phosphate regulation. Rev Endocr Metab Disord.

[6] Peacock M (2010). Calcium metabolism in health and disease. Clin J Am Soc Nephrol.

[7] Wang L, Nancollas GH, Henneman ZJ, Klein E, Weiner S (2006). Nanosized particles in bone and dissolution insensitivity of bone mineral. Biointerphases.

[8] Robertson WG, Marshall RW (1979). Calcium measurements in serum and plasma--total and ionized. CRC Crit Rev Clin Lab Sci.

[9] Pavone V, Testa G, Gioitta Iachino S, Evola FR, Avondo S, Sessa G (2015). Hypophosphatemic rickets: etiology, clinical features and treatment. Eur J Orthop Surg Traumatol.

[10] Wagner CA, Hernando N, Forster IC, Biber J (2014). The SLC34 family of sodium-dependent phosphate transporters. Pflugers Arch.

[11] Forster IC, Hernando N, Biber J, Murer H (2006). Proximal tubular handling of phosphate: A molecular perspective. Kidney Int.

[12] Shaikh A, Berndt T, Kumar R (2008). Regulation of phosphate homeostasis by the phosphatonins and other novel mediators. Pediatr Nephrol.

[13] Masi L (2011). Phosphatonins: new hormones involved in numerous inherited bone disorders. Clin Cases Miner Bone Metab.

[14] Kato S, Yoshizazawa T, Kitanaka S, Murayama A, Takeyama K (2002). Molecular genetics of vitamin D- dependent hereditary rickets. Horm Res.

[15] Wan LY, Zhang YQ, Chen MD, Liu CB, Wu JF (2015). Relationship of structure and function of DNA-binding domain in vitamin D receptor. Molecules.

[16] Glorieux FH (1997). Pseudo-vitamin D deficiency rickets. J Endocrinol.

[17] Fraser D, Kooh SW, Kind HP, Holick MF, Tanaka Y, DeLuca HF (1973). Pathogenesis of hereditary vitamin-D-dependent rickets. An inborn error of vitamin D metabolism involving defective conversion of 25-hydroxyvitamin D to 1 alpha,25-dihydroxyvitamin D. N Engl J Med.

[18] Kitanaka S, Takeyama K, Murayama A, Sato T, Okumura K, Nogami M, Hasegawa Y, Niimi H, Yanagisawa J, Tanaka T, Kato S (1998). Inactivating mutations in the 25-hydroxyvitamin D3 1alpha-hydroxylase gene in patients with pseudovitamin D-deficiency rickets. N Engl J Med.

[19] Tahir S, Demirbilek H, Ozbek MN, Baran RT, Tanriverdi S, Hussain K (2016). Genotype and Phenotype Characteristics in 22 Patients with Vitamin D-Dependent Rickets Type I. Horm Res Paediatr.

[20] Demir K, Kattan WE, Zou M, Durmaz E, BinEssa H, Nalbantoğlu Ö, Al-Rijjal RA, Meyer B, Özkan B, Shi Y (2015). Novel CYP27B1 Gene Mutations in Patients with Vitamin D-Dependent Rickets Type 1A. PLoS One.

[21] Durmaz E, Zou M, Al-Rijjal RA, Bircan I, Akçurin S, Meyer B, Shi Y (2012). Clinical and genetic analysis of patients with vitamin D-dependent rickets type 1A. Clin Endocrinol (Oxf).

[22] Alzahrani AS, Zou M, Baitei EY, Alshaikh OM, Al-Rijjal RA, Meyer BF, Shi Y (2010). A novel G102E mutation of CYP27B1 in a large family with vitamin D-dependent rickets type 1. J Clin Endocrinol Metab.

[23] Kitanaka S, Murayama A, Sakaki T, Inouye K, Seino Y, Fukumoto S, Shima M, Yukizane S, Takayanagi M, Niimi H, Takeyama K, Kato S (1999). No enzyme activity of 25-hydroxyvitamin D3 1alpha-hydroxylase gene product in pseudovitamin D deficiency rickets, including that with mild clinical manifestation. J Clin Endocrinol Metab.

[24] Wang JT, Lin CJ, Burridge SM, Fu GK, Labuda M, Portale AA, Miller WL (1998). Genetics of vitamin D 1alpha-hydroxylase deficiency in 17 families. Am J Hum Genet.

[25] Wang X, Zhang MY, Miller WL, Portale AA (2002). Novel gene mutations in patients with 1alpha-hydroxylase deficiency that confer partial enzyme activity in vitro. J Clin Endocrinol Metab.

[26] Root AW, Diamond FB, Sperling M (2014). Disorders of Mineral Homeostasis in Children and Adolescents. Pediatric Endocrinology Vol 4th edition.

[27] Lo SF (2016). Nelson Textbook of Pediatrics. 20th ed.

[28] Baştuğ F, Gündüz Z, Tülpar S, Poyrazoğlu H, Düşünsel R (2013). Urolithiasis in infants: evaluation of risk factors. World J Urol.

[29] Casella SJ, Reiner BJ, Chen TC, Holick MF, Harrison HE (1994). A possible genetic defect in 25-hydroxylation as a cause of rickets. J Pediatr.

[30] Cheng JB, Motola DL, Mangelsdorf DJ, Russell DW (2003). De-orphanization of cytochrome P450 2R1: a microsomal vitamin D 25-hydroxilase. J Biol Chem.

[31] Cheng JB, Levine MA, Bell NH, Mangelsdorf DJ, Russell DW (2004). Genetic evidence that the human CYP2R1 enzyme is a key vitamin D 25-hydroxylase. Proc Natl Acad Sci U S A.

[32] Zhu J, DeLuca HF (2012). Vitamin D 25-hydroxylase - Four decades of searching, are we there yet?. Arch Biochem Biophys.

[33] Tosson H, Rose SR (2012). Absence of mutation in coding regions of CYP2R1 gene in apparent autosomal dominant vitamin D 25-hydroxylase deficiency rickets. J Clin Endocrinol Metab.

[34] Brooks MH, Bell NH, Love L, Stern PH, Orfei E, Queener SF, Hamstra AJ, DeLuca HF (1978). Vitamin-D-dependent rickets type II. Resistance of target organs to 1,25-dihydroxyvitamin D. N Engl J Med.

[35] Marx SJ, Bliziotes MM, Nanes M (1986). Analysis of the relation between alopecia and resistance to 1,25-dihydroxyvitamin D. Clin Endocrinol (Oxf).

[36] Li M, Indra AK, Warot X, Brocard J, Messaddeq N, Kato S, Metzger D, Chambon P (2000). Skin abnormalities generated by temporally controlled RXRalpha mutations in mouse epidermis. Nature.

[37] Wan LY, Zhang YQ, Chen MD, Du YQ, Liu CB, Wu JF (2015). Relationship between Structure and Conformational Change of the Vitamin D Receptor Ligand Binding Domain in 1alpha,25-Dihydroxyvitamin D3 Signaling. Molecules.

[38] Malloy PJ, Pike JW, Feldman D (1999). The vitamin D receptor and the syndrome of hereditary 1,25-dihydroxyvitamin D-resistant rickets. Endocr Rev.

[39] Nicolaidou P, Tsitsika A, Papadimitriou A, Karantana A, Papadopoulou A, Psychou F, Liakopoulou D, Georgouli H, Kakourou T, Chrousos G (2007). Hereditary vitamin D-resistant rickets in Greek children: genotype, phenotype, and long-term response to treatment. J Pediatr Endocrinol Metab.

[40] Tiosano D, Hadad S, Chen Z, Nemirovsky A, Gepstein V, Militianu D, Weisman Y, Abrams SA (2011). Calcium absorption, kinetics, bone density, and bone structure in patients with hereditary vitamin D-resistant rickets. J Clin Endocrinol Metab.

[41] Takeda E, Yokota I, Kawakami I, Hashimoto T, Kuroda Y, Arase S (1989). Two siblings with vitamin-D-dependent rickets type II: no recurrence of rickets for 14 years after cessation of therapy. Eur J Pediatr.

[42] Malloy PJ, Zhu W, Zhao XY, Pehling GB, Feldman D (2001). A novel inborn error in the ligand-binding domain of the vitamin D receptor causes hereditary vitamin D-resistant rickets. Mol Genet Metab.

[43] al-Aqeel A, Ozand P, Sobki S, Sewairi W, Marx S (1993). The combined use of intravenous and oral calcium for the treatment of vitamin D dependent rickets type II (VDDRII). Clin Endocrinol (Oxf).

[44] Ersoy B, Kiremitci S, Isojima T, Kitanaka S (2015). Successful intermittent intravenous calcium treatment via the peripheral route in a patient with hereditary vitamin D-resistant rickets and alopecia. Horm Res Paediatr.

[45] Ma NS, Malloy PJ, Pitukcheewanont P, Dreimane D, Geffner ME, Feldman D (2009). Hereditary vitamin D resistant rickets: identification of a novel splice site mutation in the vitamin D receptor gene and successful treatment with oral calcium therapy. Bone.

[46] Celbek G, Gungor A, Albayrak H, Kir S, Guvenc SC, Aydin Y (2013). Bullous skin reaction seen after extravasation of calcium gluconate. Clin Exp Dermatol.

[47] Huang K, Malloy P, Feldman D, Pitukcheewanont P (2013). Enteral calcium infusion used successfully as treatment for a patient with hereditary vitamin D resistant rickets (HVDRR) without alopecia: a novel mutation. Gene.

[48] Akıncı A, Dündar İ, Kıvılcım M (2017). The Effectiveness of Cinacalcet as an Adjunctive Therapy for Hereditary 1,25 Dihydroxyvitamin D3-Resistant Rickets. J Clin Res Pediatr Endocrinol.

[49] Srivastava T, Alon US (2013). Cinacalcet as adjunctive therapy for hereditary 1,25-dihydroxyvitamin D-resistant rickets. J Bone Miner Res.

[50] Hewison M, Rut AR, Kristjansson K, Walker RE, Dillon MJ, Hughes MR, O’Riordan JL (1993). Tissue resistance to 1,25-dihydroxyvitamin D without a mutation of the vitamin D receptor gene. Clin Endocrinol (Oxf).

[51] Giraldo A, Pino W, Garcia-Ramirez LF, Pineda M, Iglesias A (1995). Vitamin D dependent rickets type II and normal vitamin D receptor cDNA sequence. A cluster in a rural area of Cauca, Colombia, with more than 200 affected children. Clin Genet.

[52] Chen H, Hewison M, Hu B, Adams JS (2003). Heterogeneous nuclear ribonucleoprotein (hnRNP) binding to hormone response elements: a cause of vitamin D resistance. Proc Natl Acad Sci U S A.

[53] Chen H, Hewison M, Adams JS (2006). Functional characterization of heterogeneous nuclear ribonuclear protein C1/C2 in vitamin D resistance: a novel response element-binding protein. J Biol Chem.

[54] Beck-Nielsen SS, Brock-Jacobsen B, Gram J, Brixen K, Jensen TK (2009). Incidence and prevalence of nutritional and hereditary rickets in southern Denmark. Eur J Endocrinol.

[55] Guven A, Al-Rijjal RA, BinEssa HA, Dogan D, Kor Y, Zou M, Kaya N, Alenezi AF, Hancili S, Tarim Ö, Baitei EY, Kattan WE, Meyer BF, Shi Y (2017). Mutational analysis of *PHEX*, FGF23 and CLCN5 in patients with hypophosphataemic rickets. Clin Endocrinol (Oxf).

[56] Holick MF (2007). Vitamin D deficiency. N Engl J Med.

[57] Bergwitz C, Jüppner H (2010). Regulation of phosphate homeostasis by PTH, vitamin D, and FGF23. Annu Rev Med.

[58] Prié D, Friedlander G (2010). Genetic disorders of renal phosphate transport. N Engl J Med.

[59] Li H, Martin A, David V, Quarles LD (2011). Compound deletion of Fgfr3 and Fgfr4 partially rescues the Hyp mouse phenotype. Am J Physiol Endocrinol Metab.

[60] Hu MC, Shiizaki K, Kuro-o M, Moe OW (2013). Fibroblast growth factor 23 and Klotho: physiology and pathophysiology of an endocrine network of mineral metabolism. Annu Rev Physiol.

[61] Martin A, David V, Quarles LD (2012). Regulation and function of the FGF23/klotho endocrine pathways. Physiol Rev.

[62] Razali NN, Hwu TT, Thilakavathy K (2015). Phosphatonins: new hormones involved in numerous inherited bone disorders. J Pediatr Endocrinol Metab.

[63] Turan S, Topcu B, Gökçe İ, Güran T, Atay Z, Omar A, Akçay T, Bereket A (2011). Serum alkaline phosphatase levels in healthy children and evaluation of alkaline phosphatase z-scores in different types of rickets. J Clin Res Pediatr Endocrinol.

[64] Yu ASL, Stubbs JR, Lam AQ, Lam AQ (Accessed on October 27, 2017). Waltham, MA.

[65] Barth JH, Jones RG, Payne RB (2000). Calculation of renal tubular reabsorption of phosphate: the algorithm performs better than the nomogram. Ann Clin Biochem.

[66] Payne RB (1998). Renal tubular reabsorption of phosphate (TmP/GFR): indications and interpretation. Ann Clin Biochem.

[67] Rowe PS, Oudet CL, Francis F, Sinding C, Pannetier S, Econs MJ, Strom TM, Meitinger T, Garabedian M, David A, Macher MA, Questiaux E, Popowska E, Pronicka E, Read AP, Mokrzycki A, Glorieux FH, Drezner MK, Hanauer A, Lehrach H, Goulding JN, O’Riordan JL (1997). Distribution of mutations in the PEX gene in families with X-linked hypophosphataemic rickets (HYP). Hum Mol Genet.

[68] Rowe PS (2012). Regulation of bone-renal mineral and energy metabolism: the *PHEX*, FGF23, DMP1, MEPE ASARM pathway. Crit Rev Eukaryot Gene Expr.

[69] Jonsson KB, Zahradnik R, Larsson T, White KE, Sugimoto T, Imanishi Y, Yamamoto T, Hampson G, Koshiyama H, Ljunggren O, Oba K, Yang IM, Miyauchi A, Econs MJ, Lavigne J, Jüppner H (2003). Fibroblast growth factor 23 in oncogenic osteomalacia and X-linked hypophosphatemia. N Engl J Med.

[70] Durmaz E, Zou M, Al-Rijjal RA, Baitei EY, Hammami S, Bircan I, Akçurin S, Meyer B, Shi Y (2013). Novel and de novo *PHEX* mutations in patients with hypophosphatemic rickets. Bone.

[71] Zou M, Buluş D, Al-Rijjal RA, Andıran N, BinEssa H, Kattan WE, Meyer B, Shi Y (2015). Hypophosphatemic rickets caused by a novel splice donor site mutation and activation of two cryptic splice donor sites in the *PHEX* gene. J Pediatr Endocrinol Metab.

[72] Shimada T, Kitanaka S, Muto T, Urakawa I, Yoneya T, Yamazaki Y, Okawa K, Takeuchi Y, Fujita T, Fukumoto S, Yamashita T (2002). Mutant FGF-23 responsible for autosomal dominant hypophosphatemic rickets is resistant to proteolytic cleavage and causes hypophosphatemia in vivo. Endocrinology.

[73] Econs MJ, McEnery PT (1997). Autosomal dominant hypophosphatemic rickets/osteomalacia: clinical characterization of a novel renal phosphate-wasting disorder. J Clin Endocrinol Metab.

[74] Imel EA, Hui SL, Econs MJ (2007). FGF23 concentrations vary with disease status in autosomal dominant hypophosphatemic rickets. J Bone Miner Res.

[75] Wolf M, White KE (2014). Coupling fibroblast growth factor 23 production and cleavage: iron deficiency, rickets, and kidney disease. Curr Opin Nephrol Hypertens.

[76] Farrow EG, Yu X, Summers LJ, Davis SI, Fleet JC, Allen MR, Robling AG, Stayrook KR, Jideonwo V, Magers MJ, Garringer HJ, Vidal R, Chan RJ, Goodwin CB, Hui SL, Peacock M, White KE (2011). Iron deficiency drives an autosomal dominant hypophosphatemic rickets (ADHR) phenotype in fibroblast growth factor-23 (Fgf23) knock-in mice. Proc Natl Acad Sci U S A.

[77] Imel EA, Peacock M, Gray AK, Padgett LR, Hui SL, Econs MJ (2011). Iron modifies plasma FGF23 differently in autosomal dominant hypophosphatemic rickets and healthy humans. J Clin Endocrinol Metab.

[78] Lorenz-Depiereux B, Bastepe M, Benet-Pagès A, Amyere M, Wagenstaller J, Müller-Barth U, Badenhoop K, Kaiser SM, Rittmaster RS, Shlossberg AH, Olivares JL, Loris C, Ramos FJ, Glorieux F, Vikkula M, Jüppner H, Strom TM (2006). DMP1 mutations in autosomal recessive hypophosphatemia implicate a bone matrix protein in the regulation of phosphate homeostasis. Nat Genet.

[79] Feng JQ, Ward LM, Liu S, Lu Y, Xie Y, Yuan B, Yu X, Rauch F, Davis SI, Zhang S, Rios H, Drezner MK, Quarles LD, Bonewald LF, White KE (2006). Loss of DMP1 causes rickets and osteomalacia and identifies a role for osteocytes in mineral metabolism. Nat Genet.

[80] Mäkitie O, Pereira RC, Kaitila I, Turan S, Bastepe M, Laine RC, Kröger H, Cole WG, Jüppner H (2010). Long-term clinical outcome and carrier phenotype in autosomal recessive hypophosphatemia caused by a novel DMP1 mutation. J Bone Miner Res.

[81] Levy-Litan V, Hershkovitz E, Avizov L, Leventhal N, Bercovich D, Chalifa-Caspi V, Manor E, Buriakovsky S, Hadad Y, Goding J, Parvari R (2010). Autosomal-recessive hypophosphatemic rickets is associated with an inactivation mutation in the ENPP1 gene. Am J Hum Genet.

[82] Rutsch F, Ruf N, Vaingankar S, Toliat MR, Suk A, Höhne W, Schauer G, Lehmann M, Roscioli T, Schnabel D, Epplen JT, Knisely A, Superti-Furga A, McGill J, Filippone M, Sinaiko AR, Vallance H, Hinrichs B, Smith W, Ferre M, Terkeltaub R, Nürnberg P (2003). Mutations in ENPP1 are associated with ‘idiopathic’ infantile arterial calcification. Nat Genet.

[83] Lorenz-Depiereux B, Schnabel D, Tiosano D, Häusler G, Strom TM (2010). Loss-of-function ENPP1 mutations cause both generalized arterial calcification of infancy and autosomal-recessive hypophosphatemic rickets. Am J Hum Genet.

[84] Mackenzie NC, Zhu D, Milne EM, van ‘t Hof R, Martin A, Darryl Quarles L, Millán JL, Farquharson C, MacRae VE (2012). Altered bone development and an increase in FGF-23 expression in Enpp1(-/-) mice. PLoS One.

[85] Brownstein CA, Adler F, Nelson-Williams C, Iijima J, Li P, Imura A, Nabeshima Y, Carpenter TO, Lifton RP (2008). A translocation causing increased alpha-klotho level results in hypophosphatemic rickets and hyperparathyroidism. Proc Natl Acad Sci U S A.

[86] Imura A, Tsuji Y, Murata M, Maeda R, Kubota K, Iwano A, Obuse C, Togashi K, Tominaga M, Kita N, Tomiyama K, Iijima J, Nabeshima Y, Fujioka M, Asato R, Tanaka S, Kojima K, Ito J, Nozaki K, Hashimoto N, Ito T, Nishio T, Uchiyama T, Fujimori T, Nabeshima Y (2007). Alpha-Klotho as a regulator of calcium homeostasis. Science.

[87] Kuro-o M, Matsumura Y, Aizawa H, Kawaguchi H, Suga T, Utsugi T, Ohyama Y, Kurabayashi M, Kaname T, Kume E, Iwasaki H, Iida A, Shiraki-Iida T, Nishikawa S, Nagai R, Nabeshima YI (1997). Mutation of the mouse klotho gene leads to a syndrome resembling ageing. Nature.

[88] Woudenberg-Vrenken TE, van der Eerden BC, van der Kemp AW, van Leeuwen JP, Bindels RJ, Hoenderop JG (2012). Characterization of vitamin D-deficient klotho(-/-) mice: do increased levels of serum 1,25(OH)2D3 cause disturbed calcium and phosphate homeostasis in klotho(-/-) mice?. Nephrol Dial Transplant.

[89] White KE, Cabral JM, Davis SI, Fishburn T, Evans WE, Ichikawa S, Fields J, Yu X, Shaw NJ, McLellan NJ, McKeown C, Fitzpatrick D, Yu K, Ornitz DM, Econs MJ (2005). Mutations that cause osteoglophonic dysplasia define novel roles for FGFR1 in bone elongation. Am J Hum Genet.

[90] Schwindinger WF, Francomano CA, Levine MA (1992). Identification of a mutation in the gene encoding the alpha subunit of the stimulatory G protein of adenylyl cyclase in McCune-Albright syndrome. Proc Natl Acad Sci U S A.

[91] Riminucci M PJ, Collins MT, Fedarko NS, Cherman N, Corsi A, White KE, Waguespack S, Gupta A, Hannon T, Econs MJ, Bianco P, Gehron Robey P (2003). FGF-23 in fibrous dysplasia of bone and its relationship to renal phosphate wasting. J Clin Invest.

[92] Raine J, Winter RM, Davey A, Tucker SM (1989). Unknown syndrome: microcephaly, hypoplastic nose, exophthalmos, gum hyperplasia, cleft palate, low set ears, and osteosclerosis. J Med Genet.

[93] Simpson MA, Hsu R, Keir LS, Hao J, Sivapalan G, Ernst LM, Zackai EH, Al-Gazali LI, Hulskamp G, Kingston HM, Prescott TE, Ion A, Patton MA, Murday V, George A, Crosby AH (2007). Mutations in FAM20C are associated with lethal osteosclerotic bone dysplasia (Raine syndrome), highlighting a crucial molecule in bone development. Am J Hum Genet.

[94] Simpson MA, Scheuerle A, Hurst J, Patton MA, Stewart H, Crosby AH (2009). Mutations in FAM20C also identified in non-lethal osteosclerotic bone dysplasia. Clin Genet.

[95] Wang X, Hao J, Xie Y, Sun Y, Hernandez B, Yamoah AK, Prasad M, Zhu Q, Feng JQ, Qin C (2010). Expression of FAM20C in the osteogenesis and odontogenesis of mouse. J Histochem Cytochem.

[96] Wang X, Wang S, Li C, Gao T, Liu Y, Rangiani A, Sun Y, Hao J, George A, Lu Y, Groppe J, Yuan B, Feng JQ, Qin C (2012). Inactivation of a novel FGF23 regulator, FAM20C, leads to hypophosphatemic rickets in mice. PLoS Genet.

[97] Rafaelsen SH, Raeder H, Fagerheim AK, Knappskog P, Carpenter TO, Johansson S, Bjerknes R (2013). Exome sequencing reveals FAM20c mutations associated with fibroblast growth factor 23-related hypophosphatemia, dental anomalies, and ectopic calcification. J Bone Miner Res.

[98] Takeyari S, Yamamoto T, Kinoshita Y, Fukumoto S, Glorieux FH, Michigami T, Hasegawa K, Kitaoka T, Kubota T, Imanishi Y, Shimotsuji T, Ozono K (2014). Hypophosphatemic osteomalacia and bone sclerosis caused by a novel homozygous mutation of the FAM20C gene in an elderly man with a mild variant of Raine syndrome. Bone.

[99] Kinoshita Y, Hori M, Taguchi M, Fukumoto S (2014). Functional analysis of mutant FAM20C in Raine syndrome with FGF23-related hypophosphatemia. Bone.

[100] Zonana J, Rimoin DL, Lachman RS, Cohen AH (1977). A unique chondrodysplasia secondary to a defect in chondroosseous transformation. Birth Defects Orig Artic Ser.

[101] Maroteaux P, Stanescu V, Stanescu R, Le Marec B, Moraine C, Lejarraga H (1984). Opsismodysplasia: a new type of chondrodysplasia with predominant involvement of the bones of the hand and the vertebrae. Am J Med Genet.

[102] Below JE, Earl DL, Shively KM, McMillin MJ, Smith JD, Turner EH, Stephan MJ, Al-Gazali LI, Hertecant JL, Chitayat D, Unger S, Cohn DH, Krakow D, Swanson JM, Faustman EM, Shendure J, Nickerson DA, Bamshad MJ, University of Washington Center for Mendelian Genomics (2013). Whole-genome analysis reveals that mutations in inositol polyphosphate phosphatase-like 1 cause opsismodysplasia. Am J Hum Genet.

[103] Khwaja A, Parnell SE, Ness K, Bompadre V, White KK (2015). Opsismodysplasia: Phosphate Wasting Osteodystrophy Responds to Bisphosphonate Therapy. Front Pediatr.

[104] Zeger MD, Adkins D, Fordham LA, White KE, Schoenau E, Rauch F, Loechner KJ (2007). Compound deletion of Fgfr3 and Fgfr4 partially rescues the Hyp mouse phenotype. J Pediatr Endocrinol Metab.

[105] Rafaelsen S, Johansson S, Ræder H, Bjerknes R (2016). Hereditary hypophosphatemia in Norway: a retrospective population-based study of genotypes, phenotypes, and treatment complications. Eur J Endocrinol.

[106] Bhatia V, Kulkarni A, Nair VV, Zacharin M (2013). Disorders of Mineral and Bone Metabolism. Practical Pediatric Endocrinology in a Limited Resource Setting 1st ed.

[107] Taylor A, Sherman NH, Norman ME (1995). Nephrocalcinosis in X-linked hypophosphatemia: effect of treatment versus disease. Pediatr Nephrol.

[108] Verge CF, Lam A, Simpson JM, Cowell CT, Howard NJ, Silink M (1991). Effects of therapy in X-linked hypophosphatemic rickets. N Engl J Med.

[109] Patzer L, van’t Hoff W, Shah V, Hallson P, Kasidas GP, Samuell C, de Bruyn R, Barratt TM, Dillon MJ (1999). Urinary supersaturation of calcium oxalate and phosphate in patients with X-linked hypophosphatemic rickets and in healthy schoolchildren. J Pediatr.

[110] Kooh SW, Binet A, Daneman A (1994). Nephrocalcinosis in X-linked hypophosphataemic rickets: its relationship to treatment, kidney function, and growth. Clin Invest Med.

[111] Alon US, Levy-Olomucki R, Moore WV, Stubbs J, Liu S, Quarles LD (2008). Calcimimetics as an adjuvant treatment for familial hypophosphatemic rickets. Clin J Am Soc Nephrol.

[112] Mäkitie O, Kooh SW, Sochett E (2003). Prolonged high-dose phosphate treatment: a risk factor for tertiary hyperparathyroidism in X-linked hypophosphatemic rickets. Clin Endocrinol (Oxf).

[113] Alon US, Monzavi R, Lilien M, Rasoulpour M, Geffner ME, Yadin O (2003). Hypertension in hypophosphatemic rickets--role of secondary hyperparathyroidism. Pediatr Nephrol.

[114] Novais E, Stevens PM (2006). Hypophosphatemic rickets: the role of hemiepiphysiodesis. J Pediatr Orthop.

[115] Quinlan C, Guegan K, Offiah A, Neill RO, Hiorns MP, Ellard S, Bockenhauer D, Hoff WV, Waters AM (2012). Growth in *PHEX*-associated X-linked hypophosphatemic rickets: the importance of early treatment. Pediatr Nephrol.

[116] Mäkitie O, Doria A, Kooh SW, Cole WG, Daneman A, Sochett E (2003). Early treatment improves growth and biochemical and radiographic outcome in X-linked hypophosphatemic rickets. J Clin Endocrinol Metab.

[117] Santos F, Fuente R, Mejia N, Mantecon L, Gil-Peña H, Ordoñez FA (2013). Hypophosphatemia and growth. Pediatr Nephrol.

[118] Fuente R, Gil-Peña H, Claramunt-Taberner D, Hernández O, Fernández-Iglesias A, Alonso-Durán L, Rodríguez-Rubio E, Santos F (2017). X-linked hypophosphatemia and growth. Rev Endocr Metab Disord.

[119] Rothenbuhler A, Esterle L, Gueorguieva I, Salles JP, Mignot B, Colle M, Linglart A (2017). Two-year recombinant human growth hormone (rhGH) treatment is more effective in pre-pubertal compared to pubertal short children with X-linked hypophosphatemic rickets (XLHR). Growth Horm IGF Res.

[120] Wöhrle S, Henninger C, Bonny O, Thuery A, Beluch N, Hynes NE, Guagnano V, Sellers WR, Hofmann F, Kneissel M, Graus Porta D (2013). Pharmacological inhibition of fibroblast growth factor (FGF) receptor signaling ameliorates FGF23-mediated hypophosphatemic rickets. J Bone Miner Res.

[76] Imel EA, Zhang X, Ruppe MD, Weber TJ, Klausner MA, Ito T, Vergeire M, Humphrey JS, Glorieux FH, Portale AA, Insogna K, Peacock M, Carpenter TO (2015). Prolonged Correction of Serum Phosphorus in Adults With X-Linked Hypophosphatemia Using Monthly Doses of KRN23. J Clin Endocrinol Metab.

[122] Carpenter TO, Imel EA, Ruppe MD, Weber TJ, Klausner MA, Wooddell MM, Kawakami T, Ito T, Zhang X, Humphrey J, Insogna KL, Peacock M (2014). Randomized trial of the anti-FGF23 antibody KRN23 in X-linked hypophosphatemia. J Clin Invest.

[123] Zhang B, Imel EA, Ruppe MD, Weber TJ, Klausner MA, Ito T, Vergeire M, Humphrey J, Glorieux FH, Portale AA, Insogna K, Carpenter TO, Peacock M (2016). Pharmacokinetics and pharmacodynamics of a human monoclonal anti-FGF23 antibody (KRN23) in the first multiple ascending-dose trial treating adults with X-linked hypophosphatemia. J Clin Pharmacol.

[124] Bergwitz C, Roslin NM, Tieder M, Loredo-Osti JC, Bastepe M, Abu-Zahra H, Frappier D, Burkett K, Carpenter TO, Anderson D, Garabedian M, Sermet I, Fujiwara TM, Morgan K, Tenenhouse HS, Juppner H (2006). SLC34A3 mutations in patients with hereditary hypophosphatemic rickets with hypercalciuria predict a key role for the sodium-phosphate cotransporter NaPi-IIc in maintaining phosphate homeostasis. Am J Hum Genet.

[125] Abe Y, Nagasaki K, Watanabe T, Abe T, Fukami M (2014). Association between compound heterozygous mutations of SLC34A3 and hypercalciuria. Horm Res Paediatr.

[126] Chi Y, Zhao Z, He X, Sun Y, Jiang Y, Li M, Wang O, Xing X, Sun AY, Zhou X, Meng X, Xia W (2014). A compound heterozygous mutation in SLC34A3 causes hereditary hypophosphatemic rickets with hypercalciuria in a Chinese patient. Bone.

[127] Tencza AL, Ichikawa S, Dang A, Kenagy D, McCarthy E, Econs MJ, Levine MA (2009). Hypophosphatemic rickets with hypercalciuria due to mutation in SLC34A3/type IIc sodium-phosphate cotransporter: presentation as hypercalciuria and nephrolithiasis. J Clin Endocrinol Metab.

[128] Courbebaisse M, Leroy C, Bakouh N, Salaün C, Beck L, Grandchamp B, Planelles G, Hall RA, Friedlander G, Prié D (2012). A new human NHERF1 mutation decreases renal phosphate transporter NPT2a expression by a PTH-independent mechanism. PLoS One.

[129] Prié D, Huart V, Bakouh N, Planelles G, Dellis O, Gérard B, Hulin P, Benqué-Blanchet F, Silve C, Grandchamp B, Friedlander G (2002). Nephrolithiasis and osteoporosis associated with hypophosphatemia caused by mutations in the type 2a sodium-phosphate cotransporter. N Engl J Med.

[130] Beck L, Karaplis AC, Amizuka N, Hewson AS, Ozawa H, Tenenhouse HS (1998). Targeted inactivation of Npt2 in mice leads to severe renal phosphate wasting, hypercalciuria, and skeletal abnormalities. Proc Natl Acad Sci U S A.

[131] Magen D, Berger L, Coady MJ, Ilivitzki A, Militianu D, Tieder M, Selig S, Lapointe JY, Zelikovic I, Skorecki K (2010). A loss-of-function mutation in NaPi-IIa and renal Fanconi’s syndrome. N Engl J Med.

[132] Schlingmann KP, Ruminska J, Kaufmann M, Dursun I, Patti M, Kranz B, Pronicka E, Ciara E, Akcay T, Bulus D, Cornelissen EA, Gawlik A, Sikora P, Patzer L, Galiano M, Boyadzhiev V, Dumic M, Vivante A, Kleta R, Dekel B, Levtchenko E, Bindels RJ, Rust S, Forster IC, Hernando N, Jones G, Wagner CA, Konrad M (2016). Autosomal-Recessive Mutations in SLC34A1 Encoding Sodium-Phosphate Cotransporter 2A Cause Idiopathic Infantile Hypercalcemia. J Am Soc Nephrol.

[133] Lapointe JY, Tessier J, Paquette Y, Wallendorff B, Coady MJ, Pichette V, Bonnardeaux A (2006). NPT2a gene variation in calcium nephrolithiasis with renal phosphate leak. Kidney Int.

[134] Wagner CA, Rubio-Aliaga I, Biber J, Hernando N (2014). Genetic diseases of renal phosphate handling. Nephrol Dial Transplant.

[135] Tieder M, Arie R, Modai D, Samuel R, Weissgarten J, Liberman UA (1988). Elevated serum 1,25-dihydroxyvitamin D concentrations in siblings with primary Fanconi’s syndrome. N Engl J Med.

[136] Demir K, Yildiz M, Bahat H, Goldman M, Hassan N, Tzur S, Ofir A, Magen D (2017). Clinical Heterogeneity and Phenotypic Expansion of NaPi-IIa-Associated Disease. J Clin Endocrinol Metab.

[137] Wang B, Yang Y, Friedman PA (2008). Na/H exchange regulatory factor 1, a novel AKT-associating protein, regulates extracellular signal-regulated kinase signaling through a B-Raf-mediated pathway. Mol Biol Cell.

[138] Karim Z, Gérard B, Bakouh N, Alili R, Leroy C, Beck L, Silve C, Planelles G, Urena-Torres P, Grandchamp B, Friedlander G, Prié D (2008). NHERF1 mutations and responsiveness of renal parathyroid hormone. N Engl J Med.

[139] Lloyd SE, Pearce SH, Fisher SE, Steinmeyer K, Schwappach B, Scheinman SJ, Harding B, Bolino A, Devoto M, Goodyer P, Rigden SP, Wrong O, Jentsch TJ, Craig IW, Thakker RV (1996). A common molecular basis for three inherited kidney stone diseases. Nature.

[140] Devuyst O, Thakker RV (2010). Dent’s disease. Orphanet J Rare Dis.

[141] Wrong OM, Norden AG, Feest TG (1994). Dent’s disease; a familial proximal renal tubular syndrome with low-molecular-weight proteinuria, hypercalciuria, nephrocalcinosis, metabolic bone disease, progressive renal failure and a marked male predominance. QJM.

[142] Lloyd SE, Pearce SH, Günther W, Kawaguchi H, Igarashi T, Jentsch TJ, Thakker RV (1997). Idiopathic low molecular weight proteinuria associated with hypercalciuric nephrocalcinosis in Japanese children is due to mutations of the renal chloride channel (CLCN5). J Clin Invest.

[143] Hoopes RR Jr, Raja KM, Koich A, Hueber P, Reid R, Knohl SJ, Scheinman SJ (2004). Hypophosphatemic osteomalacia and bone sclerosis caused by a novel homozygous mutation of the FAM20C gene in an elderly man with a mild variant of Raine syndrome. Kidney Int.

[144] Scheinman SJ (1998). X-linked hypercalciuric nephrolithiasis: clinical syndromes and chloride channel mutations. Kidney Int.

[145] Hoopes RR Jr, Shrimpton AE, Knohl SJ, Hueber P, Hoppe B, Matyus J, Simckes A, Tasic V, Toenshoff B, Suchy SF, Nussbaum RL, Scheinman SJ (2005). Dent Disease with mutations in OCRL1. Am J Hum Genet.

[146] Levin-Iaina N, Dinour D (2012). Renal disease with OCRL1 mutations: Dent-2 or Lowe syndrome?. J Pediatr Genet.

